# Primary thyroid diffuse large B-cell lymphoma: a case report and focused review of the literature

**DOI:** 10.1007/s00277-026-07303-7

**Published:** 2026-10-09

**Authors:** Kürsat Kirkgöz, Katja Weisel, Carsten Bokemeyer, Susanne von Kroge

**Affiliations:** Department of Oncology, Hematology and Bone Marrow Transplantation with Section Pneumology, University Cancer Center Hamburg, University Medical Center Hamburg-Eppendorf, Martinistraße 52, 20246 Hamburg, Germany

**Keywords:** Thyroid, Primary thyroid lymphoma, Diffuse large B-cell lymphoma

## Abstract

Primary thyroid diffuse large B-cell lymphoma (DLBCL) is a rare subset of primary extranodal DLBCL. Thus, there are limited evidence-based specific recommendations regarding its diagnosis and treatment. We present a case report of a patient with relapsed primary thyroid DLBCL, followed by a focused literature review covering key epidemiological, diagnostic, and therapeutic aspects. In 2017, a 74-year-old woman was diagnosed with primary thyroid non-GCB-type DLBCL (Ann Arbor IE, IPI 2) after presenting with dyspnoea, hoarseness, and cough. Treatment with R-CHOP and CNS prophylaxis achieved complete remission. Six years later, an isolated relapse occurred in the contralateral thyroid gland. The patient was treated with Pola-R-miniCHP (off-label), resulting in complete remission again. In the focused literature review core needle or surgical biopsy was found to be important for reliable histopathological characterization, while first-line treatment mainly consisted of rituximab-containing chemoimmunotherapy, occasionally combined with radiotherapy. Surgery was largely limited to diagnostic purposes. Reported 5-year overall survival rates ranged from 57% to 88%, whereas relapse or refractory disease and central nervous system involvement were rare. Registry-based studies, especially those focusing on primary extranodal DLBCL, are necessary to develop evidence-based treatment guidelines for these patients.

**Supplementary Information:**

The online version contains supplementary material available at https://doi.org/10.1007/s00277-026-07303-7.

## Introduction

With an estimated incidence of 2 cases per 1,000,000 persons per year, primary thyroid lymphoma (PTL) accounts for 0.5–5% of all thyroid malignancies and less than 2% of all extranodal non-Hodgkin lymphoma [1, 2]. Defined as an extranodal lymphoma developing in the thyroid gland with or without the involvement of regional lymph nodes or extrathyroidal tissues [1], PTL typically presents as an enlarging anterior neck mass with or without cervical lymphadenopathy, and is often associated with obstructive symptoms, whereas constitutional symptoms are only observed in up to 10–20% of patients [1, 3]. 89% of PTL develop in the context of thyroid autoimmune diseases, with Hashimoto thyroiditis (HT) being the most common risk factor associated with PTL development [1, 4]. Even though HT patients have an up to 80-fold increased risk of developing PTL, only 0.6% of patients with preexisting thyroiditis eventually develop PTL [1]. In Western nations, diffuse large B-cell lymphoma (DLBCL) is the most frequently observed type of PTL, accounting for approximately 60–70% of all PTL, followed by mucosa-associated lymphoid tissue lymphoma and follicular lymphoma [5, 6]. Common prognostic factors in DLBCL in general include advanced age, poor performance status, elevated lactate dehydrogenase levels, advanced Ann Arbor stage, and the presence of extranodal involvement [7]. Regarding primary thyroid DLBCL in particular, Yi et al. reported Myc/Bcl-2 protein co-expression, treatment modalities, and rituximab-containing therapy were independent prognostic factors [8]. However, due to the rarity of the disease and the predominantly retrospective nature of available studies, prognostic data remain limited.

Because primary thyroid DLBCL is rare, specific diagnostic and therapeutic features, particularly compared with nodal or other extranodal DLBCL, remain unknown. We present a case of a 74-year-old woman with a contralateral relapsing primary thyroid DLBCL.

## Case report

**Fig. 1:**
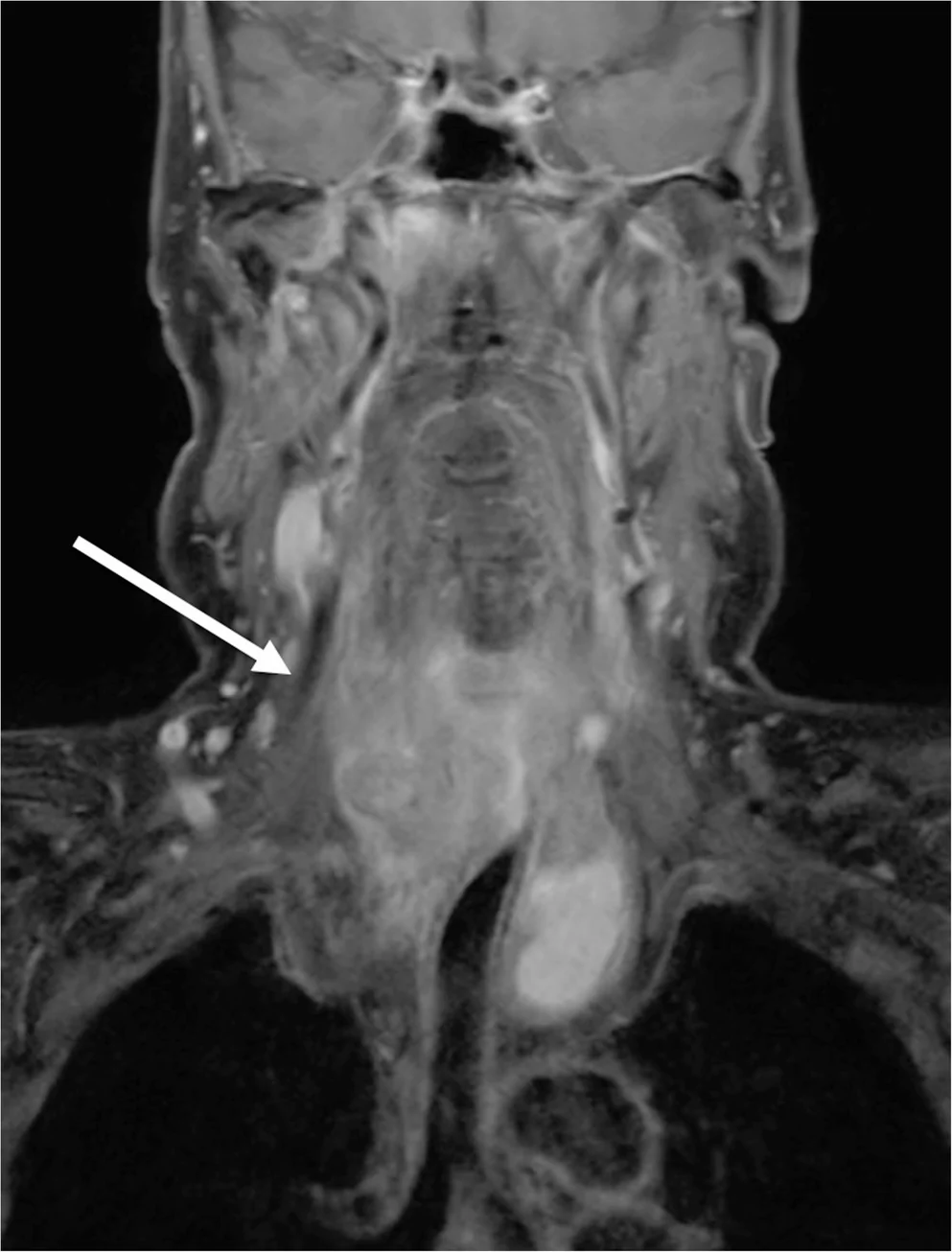
MRI from February 2017: Massive right-sided enlargement (8.5 × 8.6 × 10.6 cm) of the thyroid gland extending caudally to the aortic arch with deviation and compression of the trachea to the left

In 2017, a 74-year-old woman (ECOG 0) presented to our hospital with persistent dyspnea, hoarseness, and cough. The patient had subclinical hyperthyroidism with no history of thyroiditis. Due to an enlarged thyroid gland with a sonographic volume greater than 100 ml, causing a right deviation and compression of the trachea (Fig. 1), a pre-phase therapy with prednisone 100 mg/d was initiated, followed by a right hemithyroidectomy for suspected thyroid carcinoma. Histopathological examination led to the diagnosis of a non-GCB-type DLBCL (with immunohistochemical positivity for CD20, CD79a, and in 40% of cells Bcl-2, negativity for CD3, CD5, CD10, CD21, CD23, Bcl-6, and Cyclin D, and a Ki-67 of approximately 80%).

Complete diagnostic work-up, including computed tomography (CT) of the neck, thorax, and abdomen, as well as a bone marrow biopsy, revealed a limited-stage primary thyroid DLBCL with an international prognostic index (IPI) score of 2 (age > 60 years, LDH above the upper normal limit). The first-line treatment consisted of an anthracycline-based immunochemotherapy regimen, comprising six cycles of rituximab, doxorubicin, vincristine, cyclophosphamide, and prednisolone (R-CHOP-14 regimen), followed by two additional cycles of rituximab alone and a total of four cycles of methotrexate-containing intrathecal central nervous system prophylaxis. End-of-treatment staging showed a complete remission.

**Fig. 2:**
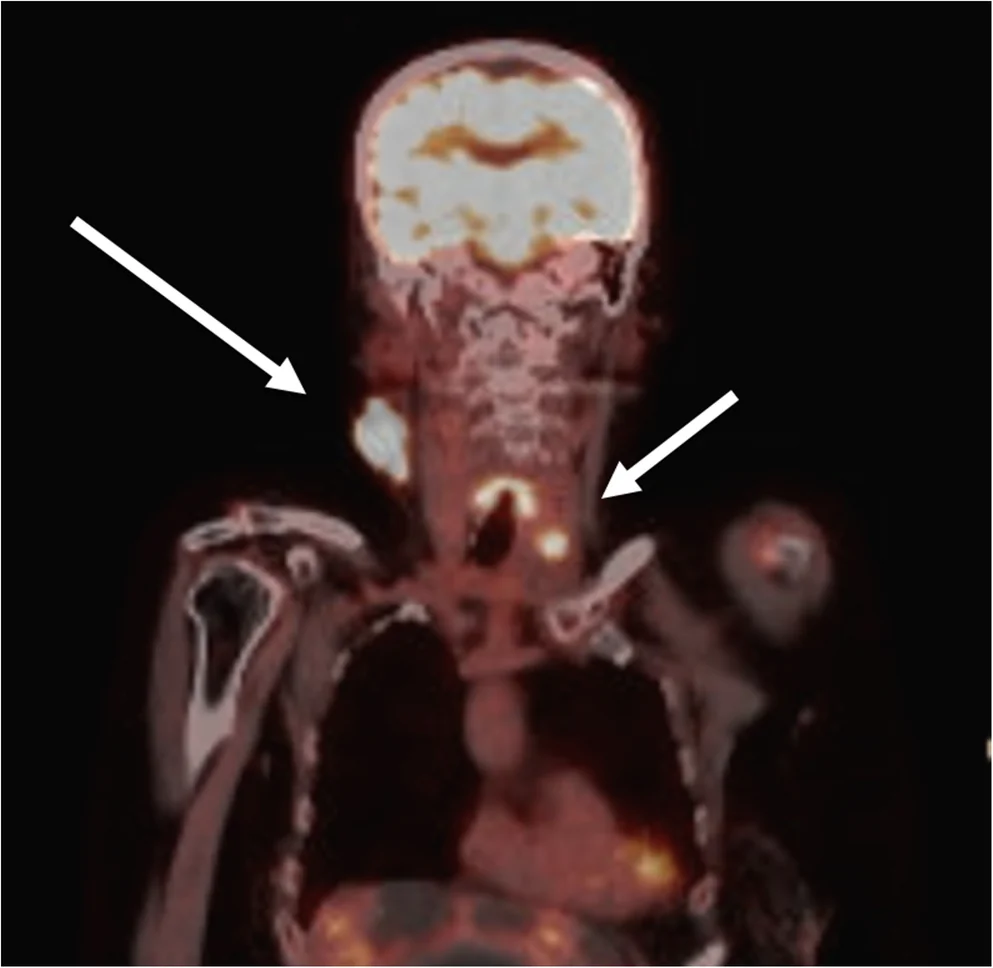
PET scan from November 2023: Increased FGD uptake in the right cervical region and left thyroid lobe

Six years after the first complete remission, the patient was admitted with suspected relapse, presenting with a firm, painless, rapidly enlarging mass in the right cervical region. 18 F-fluorodeoxyglucose (FDG)-positron emission tomography (PET)/CT revealed intense metabolic activity in the right cervical subcutaneous tissue and focally increased uptake in the remaining left thyroid gland (Fig. 2). Histopathological workup of the mass on the right cervical confirmed the diagnosis of a non-GCB-type DLBCL. The evaluation revealed a limited-stage late relapse of DLBCL, with an IPI score of 1. However, a fine-needle aspiration (FNA) could reveal that the elevated metabolic activity in the remaining left thyroid gland was not due to lymphoma but rather to focal autonomy. Considering the patient’s excellent physical status (ECOG 0), she received an immunochemotherapy regimen consisting of six cycles of polatuzumab-vedotin, rituximab, cyclophosphamide, doxorubicin, and prednisolone (Pola-R-miniCHP) on an individualized basis as an off-label use. The cumulative anthracycline dose was not exceeded. End-of-treatment staging showed a complete remission according to Lugano criteria.

## A focused review of the literature

### Definition, epidemiology, and risk factors of primary thyroid DLBCL

In the following, primary thyroid DLBCL is defined as an extranodal DLBCL involving the thyroid gland, with or without regional nodal involvement (Ann Arbor stage I A/B E or II A/B E). Other than in nodal DLBCL, females are distinctly more frequently affected by primary thyroid DLBCL, with up to 68–75% (male-to-female ratio of approximately 1:3) [9]. In addition, reliable data on differences regarding other epidemiological factors like age, race, or global distribution in patients with primary thyroid DLBCL compared to nodal DLBCL are lacking.

Since thyroid tissue lacks lymphoid tissue, it has been hypothesized that PTL arises from chronic thyroid inflammation. Indeed, 86–89% of PTL develop in the context of thyroid autoimmune diseases, with HT being the most common risk factor associated with the development of PTL, with an up to 80-fold increased risk [1, 4]. However, although PTL is strongly associated with HT, regarding primary thyroid DLBCL, Yi et al. reported preexisting HT in only 38% of patients (22 of 58 cases) [8].

### Diagnostic workup of primary thyroid DLBCL

Based on the clinical presentation, as an enlarging anterior neck mass with or without cervical lymphadenopathy accompanied by possible obstructive symptoms, a clear preoperative differentiation from other thyroid neoplasms is difficult [10]. Hyperthyroidism is present in 7–36% of patients, while hypothyroidism occurs in 27–81% [11]. Most commonly, ultrasound (US) shows a hypoechoic mass, with echogenicity less than that of the adjacent neck musculature, combined with hypervascularity and a characteristically undifferentiated outline. In contrast to other thyroid malignancies, calcifications are uncommon in the US examination [2, 12]. For contrast-enhanced US to differentiate PTL from nodular HT, a sensitivity and specificity of 84% and 88%, respectively, were reported [13].

On magnetic resonance imaging (MRI), PTL exhibits iso- or hyperintensities in T1- and T2-weighted images. Since MRI has a high contrast resolution for soft tissues, it can clearly show the uninvolved thyroid tissue. Thus, MRI may be more sensitive than CT in detecting extrathyroidal involvement [14, 15]. Recently, PET-CT has become essential in the detection and staging of DLBCL. 18 F-FDG PET-CT images indicate the metabolic activity, by showing increased uptake of 18 F-FDG in the thyroid tissue, and help to stage, restage, or evaluate the response to treatment [16–18]. 

Histopathological tissue examination is the gold standard for diagnosing PTL. The FNA method could be considered as an initial diagnostic tool [19]. However, at least approximately 10% of cases can be missed with FNA [20], even though the accuracy of FNA is increasing with the help of flow cytometry or immunohistochemical staining [21]. Based on a systematic review and meta-analysis, low sensitivity rates of approximately 50% were reported for detecting DLBCL [22]. Core needle biopsy (CNB) could obtain more tissue, with a higher diagnostic sensitivity than FNA. However, when PTL is associated with airway compromise, CNB may exacerbate dyspnoea or even cause asphyxia due to secondary haemorrhage and airway oedema [23, 24]. On the other hand, based on a meta-analysis, the need for additional diagnostic surgery after CNB was significantly lower than after FNA [21, 25, 26]. However, some prefer an open biopsy, especially when the patient has obstructive symptoms, which could be reduced by surgical intervention [27].

### Treatment and prognosis of thyroid diffuse large B-cell lymphoma - Previously untreated thyroid diffuse large B-cell lymphoma

Up to now, the optimal treatment of newly diagnosed primary thyroid DLBCL, particularly the role of surgery, has been discussed controversially [8, 28–30]. Although surgical excision, including lobectomy, hemithyroidectomy, and thyroidectomy, might be useful for diagnostic workup, Yi et al. observed significantly impaired five-year overall- and progression-free survival (PFS) of 35% and 32%, respectively, for patients receiving surgery alone compared to 61% and 54%, and 81% and 78%, in patients treated with chemotherapy alone or combined chemo- and radiotherapy [8]. Notably, five-year overall survival (OS) was approximately 79% and 50% for patients with and without a rituximab-containing systemic therapy, respectively, indicating that rituximab-containing therapies improve the survival rates of primary thyroid DLBCL patients [8]. However, there are also conflicting findings reporting that surgery alone is not inferior to surgery combined with either radio- or chemotherapy or radio- and/or chemotherapy without surgery by observing no significant differences in disease-specific survival (DSS) in patients with stage IE primary thyroid DLBCL [30]. The results reported by Tang et al. do not provide a clear presentation of the DSS for chemotherapy alone, nor do the authors provide details on the subset of patients receiving a CD20-directed treatment combination, thus limiting the interpretation of the results [30]. Another study did not report an improvement in lymphoma-related death rates by adding rituximab to CHOP. However, Olszewski et al. were unable to provide details on potential multimodal treatment [31].

A retrospective study by the International Extranodal Lymphoma Study Group and Intergruppo Italiano Linfomi including 48 patients diagnosed between 1985 and 2006 with primary thyroid DLBCL showed significantly longer PFS in patients receiving combined therapies compared to those receiving chemotherapy or radiotherapy (RT) alone (five-year PFS 64% vs. 21%) [32]. However, no significant OS difference was observed [32]. A comparably extensive international Cancer Network study (1986–2006) demonstrated significantly superior survival in patients with aggressive PTL treated with combined chemo- and radiotherapy compared to single modality treatment with chemotherapy or RT (5-year OS single- vs. multimodality 60 vs. 90%, *p* = 0.005, 5-year DSS single- vs. multimodality 54 vs. 89%, *p* = 0.001). However, limiting the transition of these results into the modern treatment era, only a minority of patients received rituximab [1]. Similarly, Ermann et al. reported a significant survival benefit in patients treated multimodally (chemo- and radiotherapy) compared to those treated with chemotherapy alone (5-year OS 91% vs. 83%, *p* = 0.001) [33].

As the International Lymphoma Radiation Oncology Group suggests, decision-making regarding consolidative RT in DLBCL should consider initial tumor size, response to systemic therapy, and potential RT morbidity. In the case of PET-guided consolidative RT of thyroid lymphoma, the clinical target volume should include the whole thyroid and should consist of the prechemotherapy or preresection gross tumor volume. For planning 3D conformal RT or IMRT is recommended [34]. However, there is little evidence specific to RT in PTL, and data from thyroid carcinomas are not comparable, as the radiation methods used in thyroid carcinomas differ from those used in lymphoma. A case series reported on nine patients who received RT for PTL (with six of them having DLBCL), treated with a median radiation dose of 40 Gy [35]. Acute-phase radiation toxicity consisted of grade 1 dermatitis and mucositis. In patients whose upper mediastinum was irradiated, late-phase radiation toxicity emerged in the form of grade 1 pneumonitis, while no grade 2 or higher toxicity was reported. Three out of eight patients developed hypothyroidism after RT [35].

In summary, although several retrospective single- and multicenter studies and registry analyses on PTL have been published, few focus on the survival of primary thyroid DLBCL patients [8, 9, 31–33, 36–40]. A comparison focusing on the prognostic impact of the site of extranodal DLBCL involvement did not observe a survival disadvantage for patients with thyroid involvement treated with R-CHOP. However, the authors did not provide further details on the 44 patients investigated [37].

Table S1 presents an overview of the most relevant registry-based, retrospective, multicenter, and single-center studies investigating the treatment and outcomes of patients with thyroid DLBCL, which provide data on at least 20 patients. A total of 17 studies were listed. Of those, eleven were conducted during the rituximab era. Considering only the results of studies including patients after 2003, 5-year OS rates span between 57 and 88% [8, 10, 33, 38, 39].

Based on the data presented and the absence of prospective or randomized trials, there is currently not enough evidence to support varying the treatment of primary thyroid DLBCL from the standard treatments used for DLBCL in general or other primary extranodal DLBCL. However, it is important to note that no specific guidelines for extranodal DLBCL address the subset of primary thyroid DLBCL [41, 42].

### Treatment and prognosis of thyroid diffuse large B-cell lymphoma - Refractory or relapsed primary thyroid diffuse large B-cell lymphoma

Currently, there is limited data on treating relapsed or refractory (r/r) primary thyroid DLBCL. Yi and colleagues reported a 17% relapse rate in patients with primary thyroid DLBCL. However, in the cohort reported by Yi et al., treatments varied; 15 of 58 patients had only surgery, while 17 of 58 received chemotherapy alone without rituximab [8]. Thus, the relapse rate for primary thyroid DLBCL might be significantly lower when considering current standard first-line treatments for DLBCL. Similarly, Watanabe et al. observed a 16% relapsed or refractory disease rate among patients with aggressive PTL, including those with DLBCL and DLBCL with MALT [4]. However, since the cohort was treated from 1990 to 2004, prior to the rituximab era, current relapse rates are probably lower [4]. In another study of a cohort treated during the rituximab era, Watanabe and colleagues reported high response and OS-rates in elderly patients with primary thyroid DLBCL treated with R-CHOP [39].

In conclusion, treatment recommendations do not currently differ from those for r/r DLBCL in general and include chemotherapy-free or -containing B-cell-directed treatment approaches [42].

### Risk of central nervous relapse

With an overall two-year risk of 4–5%, secondary involvement of the central nervous system (CNS) is a rare complication of DLBCL, typically occurring within the first year (median onset: approximately 6–9 months) after the initial diagnosis. Secondary CNS lymphoma is associated with a poor prognosis, with a median OS of approximately 17 months [43–45]. However, so far, there is no specific data on the risk of secondary CNS relapse or particular treatment recommendations for PTL patients. Yi et al. reported two cases of brain involvement at relapse [8]. Due to the lack of evidence-based data indicating an increased risk of CNS relapse in PTL patients, cerebrospinal fluid puncture and prophylactic measures should be guided by established risk prediction models, such as the CNS-IPI, and decided on an individual basis [42, 43].

## Conclusion

Available studies are partly focused on PTL in general, while long-term analyses still contain pre-rituximab data or do not precisely define thyroid DLBCL, thereby complicating study comparisons and the derivation of evidence-based recommendations.

Further extensive retrospective and prospective studies focusing particularly on primary thyroid DLBCL are needed to establish evidence-based recommendations for the optimal treatment of these patients.

## Supplementary Information

Below is the link to the electronic supplementary material.

Supplementary file 1 (DOCX 52.0 KB)

Table S1 (DOCX 105 KB)

## Data Availability

No datasets were generated or analysed during the current study.
