# Supplementary material for: Primary thyroid diffuse large B-cell lymphoma: a case report and focused review of the literature
Source: Ann Hematol. 2026 Oct 9;105(11):447. doi: 10.1007/s00277-026-07303-7 (PMC13652935; doi:10.1007/s00277-026-07303-7)
Supplement: Supplementary file 1 [file 277_2026_7303_MOESM1_ESM.docx]

Table S1: Overview of registry-based, retrospective multicenter, and single-center studies investigating treatment and outcomes of patients with thyroid diffuse large B-cell lymphoma (DLBCL). Only studies including at least 20 patients with thyroid DLBCL are listed. Studies are presented in alphabetical order.

| **Author,**  **Year** | **Number of included patients (n)** | **Study period** | **Definition of Primary thyroid DLBCL** | **Rate of thyroid DLBCL (%)** | **Treatment approach (n)** | **Rate of refractor or relapsed disease (%)** | **5-year OS (%)** | **Comments** | **Reference** |
| --- | --- | --- | --- | --- | --- | --- | --- | --- | --- |
| **Registry-based studies** | | | | | | | | | |
| Castillo et al., 2014 | 311  (thyroid DLBCL) | 2004-2009 | No specific definition available | 100 | NA | NA | NA | No association with OS in multivariate analysis | [41] |
| Chen et al., 2018 | 1108 (PTL) | 2005-2017 | NA | 77 | NA | NA | NA | NA | [47] |
| Chen et al., 2025 | 162  (thyroid DLBCL) | 2002-2022 | No specific definition available | 100 | NA | NA | NA | No association with OS in multivariate analysis | [37] |
| Costa et al., 2013 | 487 (thyroid DLBCL) | 2001-2009 | Cases not restricted to Ann Arbor stage I/II | 100 | Surgery alone (89)  Surgery + RT (116)  Surgery + CT (unknown)  CT alone (unknown)  RT alone (88)  CT+ RT (unknown) | NA | Approximately 70% | No information on CT | [9] |
| Ermann et al., 2023 | 1059  (thyroid DLBCL) | 2004-2015 | Extranodal DLBCL with limited disease stage (Ann Arbor stage I/II) | 100 | Surgery alone (0)  Surgery + RT (0)  Surgery + CT (0)  CT alone (460)  RT alone (0)  CT+ RT (599) | NA | 88 | Improved OS in multimodal treatment vs. single modality | [34] |
| Olzewski et al, 2014 | 155 (thyroid DLBCL) | 1996-2009 | NA | 100 | CHOP vs. R-CHOP | NA | 69 | No lower rated of lymphoma-related deaths between both groups | [32] |
| Toloza et al., 2025 | 2465 (PTL) | 1975-2021 | NA | 63 | Surgery +/- RT (390)  Surgery +/- CT (774) | NA | 73 | NA | [6] |
| **Multicentric retrospective studies** | | | | | | | | | |
| Chai et al., 2015 | 38 (PTL) | 2000-2013 | NA | 42 | NA | NA | 88 | Small sample sizes | [48] |
| Hirokawa et al., 2020 | 153 (PTL) | 2009-2019 | NA | 39 | NA | NA | NA | NA | [49] |
| Mian et al., 2011 | 48 (thyroid DLBCL) | 1985-2006 | Lymphoma  which presented primarily as a thyroid gland  tumor without any signs at any other site, excluding  satellite lymph nodes. | 100 | NA | 1 | 52 | NA | [33] |
| Onal et al., 2011 | 87 (PTL) | 1986-2006 | NA | 60^1^ | Surgery alone (0)  Surgery + RT (0)  Surgery + CT (0)  CT alone (35)^1^  RT alone (15)^1^  CT+ RT (24)^1^ | NA | 73 | NA | [1] |
| Takahashi et al., 2012 | 44 (thyroid DLBCL) | 2003-2006 | No specific definition available | 100 | R-CHOP | NA | NA | No association with OS in univariate analysis | [38] |
| Yi et al., 2020 | 58 (thyroid DLBCL) | 2007-2017 | „Primary thyroid DLBCL developed in the thyroid gland with or  without the involvement of regional lymph nodes and without  metastasis to other visceral tissues“ | 100 | Surgery alone (15)  Surgery + RT (0)  Surgery + CT (0)  CT alone (18)  RT alone (0)  CT + RT (25) | 17 | 73 | NA | [8] |
| **Single-center retrospective studies** | | | | | | | | | |
| Alzouebi et al., 2012 | 70 (PTL) | 1970-2010 | NA | 71 | NA | NA | 45 | NA | [50] |
| Shi et al., 2019 | 28 (thyroid DLBCL) | 2006-2012 | Extrabnodal DLBCL in general was defined as DLBCL arising  primarily from sites other than the lymphonodus,  Waldeyer’s ring, spleen, or mediastinum. | 100 | NA | NA | 57 | NA | [39] |
| Watanabe et al, 2011 | 171 (PTL) | 1990-2004 | NA | 43 | Surgery alone (0)  Surgery + RT (0)  Surgery + CT (0)  CT alone (5)^1^  RT alone (23)^1^  CT + RT (54)^1^ | 16 | NA | NA | [4] |
| Watanabe et al., 2014 | 43 (thyroid DLBCL) | 2005-2011 | Stage IE or stage IIE aggressive B-cell PTL | 100 | NA | 5 | 87 | NA | [40] |

CT: chemotherapy; DLBCL: Diffuse large B-cell lymphoma; NA: not applicable; PTL: Primary thyroid lymphoma; RT: Radiotherapy,

^1^ Reported only for aggressive lymphoma in general.
