# Supplementary material for: Primary thyroid diffuse large B-cell lymphoma: a case report and focused review of the literature
Source: Ann Hematol. 2026 Oct 9;105(11):447. doi: 10.1007/s00277-026-07303-7 (PMC13652935; doi:10.1007/s00277-026-07303-7)
Supplement: Supplementary file 2 [file 277_2026_7303_MOESM2_ESM.docx]

Table S1: Overview of registry-based, retrospective multicenter, and single-center studies investigating treatment and outcomes of patients with thyroid diffuse large B-cell lymphoma (DLBCL). Only studies including at least 20 patients with thyroid DLBCL are listed. Studies are presented in alphabetical order.

| **Author,**  **Year** | **Number of included patients (n)** | **Study period** | **Definition of Primary thyroid DLBCL** | **Rate of thyroid DLBCL (%)** | **Treatment approach (n)** | **Rate of refractor or relapsed disease (%)** | **5-year OS (%)** | **Comments** | **Reference** |
| --- | --- | --- | --- | --- | --- | --- | --- | --- | --- |
| **Registry-based studies** | | | | | | | | | |
| Castillo et al., 2014 | 311  (thyroid DLBCL) | 2004-2009 | No specific definition available | 100 | NA | NA | NA | No association with OS in multivariate analysis | [1] |
| Chen et al., 2018 | 1108 (PTL) | 2005-2017 | NA | 77 | NA | NA | NA | NA | [2] |
| Chen et al., 2025 | 162  (thyroid DLBCL) | 2002-2022 | No specific definition available | 100 | NA | NA | NA | No association with OS in multivariate analysis | [3] |
| Costa et al., 2013 | 487 (thyroid DLBCL) | 2001-2009 | Cases not restricted to Ann Arbor stage I/II | 100 | Surgery alone (89)  Surgery + RT (116)  Surgery + CT (unknown)  CT alone (unknown)  RT alone (88)  CT+ RT (unknown) | NA | Approximately 70% | No information on CT | [4] |
| Ermann et al., 2023 | 1059  (thyroid DLBCL) | 2004-2015 | Extranodal DLBCL with limited disease stage (Ann Arbor stage I/II) | 100 | Surgery alone (0)  Surgery + RT (0)  Surgery + CT (0)  CT alone (460)  RT alone (0)  CT+ RT (599) | NA | 88 | Improved OS in multimodal treatment vs. single modality | [5] |
| Olzewski et al, 2014 | 155 (thyroid DLBCL) | 1996-2009 | NA | 100 | CHOP vs. R-CHOP | NA | 69 | No lower rated of lymphoma-related deaths between both groups | [6] |
| Toloza et al., 2025 | 2465 (PTL) | 1975-2021 | NA | 63 | Surgery +/- RT (390)  Surgery +/- CT (774) | NA | 73 | NA | [7] |
| **Multicentric retrospective studies** | | | | | | | | | |
| Chai et al., 2015 | 38 (PTL) | 2000-2013 | NA | 42 | NA | NA | 88 | Small sample sizes | [8] |
| Hirokawa et al., 2020 | 153 (PTL) | 2009-2019 | NA | 39 | NA | NA | NA | NA | [9] |
| Mian et al., 2011 | 48 (thyroid DLBCL) | 1985-2006 | Lymphoma  which presented primarily as a thyroid gland  tumor without any signs at any other site, excluding  satellite lymph nodes. | 100 | NA | 1 | 52 | NA | [10] |
| Onal et al., 2011 | 87 (PTL) | 1986-2006 | NA | 60^1^ | Surgery alone (0)  Surgery + RT (0)  Surgery + CT (0)  CT alone (35)^1^  RT alone (15)^1^  CT+ RT (24)^1^ | NA | 73 | NA | [11] |
| Takahashi et al., 2012 | 44 (thyroid DLBCL) | 2003-2006 | No specific definition available | 100 | R-CHOP | NA | NA | No association with OS in univariate analysis | [12] |
| Yi et al., 2020 | 58 (thyroid DLBCL) | 2007-2017 | „Primary thyroid DLBCL developed in the thyroid gland with or  without the involvement of regional lymph nodes and without  metastasis to other visceral tissues“ | 100 | Surgery alone (15)  Surgery + RT (0)  Surgery + CT (0)  CT alone (18)  RT alone (0)  CT + RT (25) | 17 | 73 | NA | [13] |
| **Single-center retrospective studies** | | | | | | | | | |
| Alzouebi et al., 2012 | 70 (PTL) | 1970-2010 | NA | 71 | NA | NA | 45 | NA | [14] |
| Shi et al., 2019 | 28 (thyroid DLBCL) | 2006-2012 | Extrabnodal DLBCL in general was defined as DLBCL arising  primarily from sites other than the lymphonodus,  Waldeyer’s ring, spleen, or mediastinum. | 100 | NA | NA | 57 | NA | [15] |
| Watanabe et al, 2011 | 171 (PTL) | 1990-2004 | NA | 43 | Surgery alone (0)  Surgery + RT (0)  Surgery + CT (0)  CT alone (5)^1^  RT alone (23)^1^  CT + RT (54)^1^ | 16 | NA | NA | [16] |
| Watanabe et al., 2014 | 43 (thyroid DLBCL) | 2005-2011 | Stage IE or stage IIE aggressive B-cell PTL | 100 | NA | 5 | 87 | NA | [17] |

CT: chemotherapy; DLBCL: Diffuse large B-cell lymphoma; NA: not applicable; PTL: Primary thyroid lymphoma; RT: Radiotherapy,

^1^ Reported only for aggressive lymphoma in general.

**Refrences:**

1. Castillo JJ, Winer ES, Olszewski AJ. Sites of extranodal involvement are prognostic in patients with diffuse large B-cell lymphoma in the rituximab era: an analysis of the Surveillance, Epidemiology and End Results database. Am J Hematol [Internet]. Am J Hematol; 2014 [cited 2025 Aug 27];89:310–4. https://doi.org/10.1002/AJH.23638

2. Chen E, Wu Q, Jin Y, Jin W, Cai Y, Wang Q, et al. Clinicopathological characteristics and prognostic factors for primary thyroid lymphoma: report on 28 Chinese patients and results of a population-based study. Cancer Manag Res [Internet]. Cancer Manag Res; 2018 [cited 2025 Aug 29];10:4411–9. https://doi.org/10.2147/CMAR.S155170

3. Chen SY, Xu PP, Feng R, Cui GH, Wang L, Cheng S, et al. Extranodal diffuse large B-cell lymphoma: Clinical and molecular insights with survival outcomes from the multicenter EXPECT study. Cancer Commun (Lond) [Internet]. Cancer Commun (Lond); 2025 [cited 2025 Aug 28];45. https://doi.org/10.1002/CAC2.70033

4. Costa LJ, Xavier AC. Features and outcomes of diffuse large B-cell lymphoma of the thyroid in a large contemporary cohort. Leuk Lymphoma [Internet]. Leuk Lymphoma; 2014 [cited 2025 Aug 27];55:283–7. https://doi.org/10.3109/10428194.2013.802317

5. Ermann DA, Vardell VA, Shah H, Fitzgerald L, Tao R, Gaffney DK, et al. Survival Outcomes of Limited-Stage Diffuse Large B-Cell Lymphoma Treated With Radiation Therapy. Clin Lymphoma Myeloma Leuk [Internet]. Clin Lymphoma Myeloma Leuk; 2024 [cited 2025 Aug 29];24:94-104.e6. https://doi.org/10.1016/J.CLML.2023.09.006

6. Olszewski AJ, Winer ES, Castillo JJ. Improved survival with rituximab-based chemoimmunotherapy in older patients with extranodal diffuse large B-cell lymphoma. Leuk Res. Pergamon; 2014;38:866–73. https://doi.org/10.1016/J.LEUKRES.2014.04.009

7. Toloza FJK, Gubbi S, Klubo-Gwiezdzinska J. Trends in Survival and Factors Associated with Survival in Primary Thyroid Lymphoma: A SEER-Based Analysis (1975-2021). Thyroid [Internet]. Thyroid; 2025 [cited 2025 Aug 29]; https://doi.org/10.1177/10507256251363141

8. Chai YJ, Hong JH, Koo DH, Yu HW, Lee JH, Kwon H, et al. Clinicopathological characteristics and treatment outcomes of 38 cases of primary thyroid lymphoma: a multicenter study. Ann Surg Treat Res [Internet]. Ann Surg Treat Res; 2015 [cited 2025 Aug 29];89:295–9. https://doi.org/10.4174/ASTR.2015.89.6.295

9. Hirokawa M, Suzuki A, Hashimoto Y, Satoh S, Canberk S, Jhuang JY, et al. Prevalence and diagnostic challenges of thyroid lymphoma: a multi-institutional study in non-Western countries. Endocr J [Internet]. Endocr J; 2020 [cited 2025 Aug 29];67:1085–91. https://doi.org/10.1507/ENDOCRJ.EJ20-0202

10. Mian M, Gaidano G, Conconi A, Tsang R, Gospodarowicz MK, Rambaldi A, et al. High response rate and improvement of long-term survival with combined treatment modalities in patients with poor-risk primary thyroid diffuse large B-cell lymphoma: an International Extranodal Lymphoma Study Group and Intergruppo Italiano Linfomi study. Leuk Lymphoma [Internet]. Leuk Lymphoma; 2011 [cited 2025 Aug 30];52:823–32. https://doi.org/10.3109/10428194.2011.555888

11. Onal C, Li YX, Miller RC, Poortmans P, Constantinou N, Weber DC, et al. Treatment results and prognostic factors in primary thyroid lymphoma patients: A Rare Cancer Network study. Annals of Oncology [Internet]. Oxford University Press; 2011 [cited 2025 Aug 27];22:156–64. https://doi.org/10.1093/annonc/mdq310

12. Takahashi H, Tomita N, Yokoyama M, Tsunoda S, Yano T, Murayama K, et al. Prognostic impact of extranodal involvement in diffuse large B-cell lymphoma in the rituximab era. Cancer. 2012;118:4166–72. https://doi.org/10.1002/CNCR.27381

13. Yi J, Yi P, Wang W, Wang H, Wang X, Luo H, et al. A Multicenter Retrospective Study of 58 Patients With Primary Thyroid Diffuse Large B Cell Lymphoma. Front Endocrinol (Lausanne). Frontiers Media S.A.; 2020;11. https://doi.org/10.3389/FENDO.2020.00542/FULL

14. Alzouebi M, Goepel JR, Horsman JM, Hancock BW. Primary thyroid lymphoma: the 40 year experience of a UK lymphoma treatment centre. Int J Oncol [Internet]. Int J Oncol; 2012 [cited 2025 Aug 30];40:2075–80. https://doi.org/10.3892/IJO.2012.1387

15. Shi Y, Han Y, Yang J, Liu P, He X, Zhang C, et al. Clinical features and outcomes of diffuse large B-cell lymphoma based on nodal or extranodal primary sites of origin: Analysis of 1,085 WHO classified cases in a single institution in China. Chin J Cancer Res [Internet]. Chin J Cancer Res; 2019 [cited 2025 Aug 28];31:152–61. https://doi.org/10.21147/J.ISSN.1000-9604.2019.01.10

16. Watanabe N, Noh JY, Narimatsu H, Takeuchi K, Yamaguchi T, Kameyama K, et al. Clinicopathological features of 171 cases of primary thyroid lymphoma: a long-term study involving 24 553 patients with Hashimoto’s disease. Br J Haematol [Internet]. John Wiley & Sons, Ltd; 2011 [cited 2025 Aug 27];153:236–43. https://doi.org/10.1111/J.1365-2141.2011.08606.X

17. Watanabe N, Narimatsu H, Noh JY, Kunii Y, Mukasa K, Matsumoto M, et al. Rituximab-including combined modality treatment for primary thyroid lymphoma: an effective regimen for elderly patients. Thyroid [Internet]. Thyroid; 2014 [cited 2025 Aug 29];24:994–9. https://doi.org/10.1089/THY.2013.0523
